# Heavy metal exposure linked to metabolic syndrome in Korean male firefighters: FRESH cohort cross-sectional analysis

**DOI:** 10.1038/s41598-023-41158-6

**Published:** 2023-08-28

**Authors:** Jee Eun Choi, Mun-Joo Bae, Mi-Ji Kim, Sung Soo Oh, Ki Soo Park, Chan Joo Lee, Sungha Park, Sang-Baek Koh, Jaelim Cho, Changsoo Kim

**Affiliations:** 1 Department of Public Health, Yonsei University College of Medicine, Seoul, Korea; 2 Institute of Human Complexity and Systems Science, Yonsei University, Incheon, Korea; 3 Institute of East West Studies, Yonsei University, Seoul, Korea; 4 Department of Occupational and Environmental Health, Yonsei University Graduate School, Seoul, Korea; 5 Department of Preventive Medicine and Institute of Health Science, Gyeongsang National University College of Medicine, Jinju, Korea; 6 Department of Occupational and Environmental Medicine, Yonsei University Wonju College of Medicine, Wonju, Korea; 7 Division of Cardiology, Yonsei Cardiovascular Hospital, Yonsei University College of Medicine, Seoul, Korea; 8 Department of Preventive Medicine, Yonsei University Wonju College of Medicine, Wonju, Korea; 9 Department of Preventive Medicine, Yonsei University College of Medicine, 50-1 Yonsei-Ro, Seodaemun-Gu, Seoul, Republic of Korea; 10 Institute for Environmental Research, Yonsei University College of Medicine, Seoul, Korea; 11 Department of Public Health, Yonsei University Graduate School, Seoul, Korea

**Keywords:** Cardiology, Health occupations

## Abstract

This study aimed to identify the relationship between blood lead and Cadmium (Cd) concentrations and metabolic syndromes (MetS), including its components (central obesity, hypertriglyceridemia, low high-density lioioritein, hypertension, and hyperglycemia) among Korean firefighters. A total of 965 firefighters of the Enhancement of Safety and Health cohort were analyzed in this study. MetS was defined according to the 2005 revised National Cholesterol Education Program-Adult Treatment Panel III criteria and the Korean Society for the Study of Obesity criteria for waist circumference. The collected data were analyzed using a logistic regression model. Of the 965 participants, 190 (19.7%) had MetS. After adjusting for age, body mass index, smoking, drinking, exercise, shift duty, and main duty position, the Cd level was significantly associated with an increased risk of MetS in the Korean firefighter population (odds ratio [OR] = 1.62, 95% confidence interval [CI] 1.07, 2.46). This association was significant among non-smokers and ex-smokers (OR = 1.58, 95% CI 1.03, 2.43), non-drinkers and ex-drinkers (OR = 1.77, 95% CI 1.06, 2.94), firefighters aged 40 year or older (OR = 1.77, 95% CI 1.10, 2.86), and office administrators (OR = 3.85, 95% CI 1.42, 10.39). This outcome suggests that exposure to Cd is likely to increase risk of MetS among firefighters.

## Introduction

Firefighters are an occupational group easily exposed to harmful pollutants, including heavy metals, which can lead to adverse health effects, including cardiovascular disease (CVD)^1–5^. CVD is known to be the primary cause of death in the line of duty among American firefighters, accounting for 43.7% of all duty-related mortality in American firefighters^2,3^. Noh et al. found that Korean firefighters were at a 1.3 fold increased risk of death or hospitalization from major adverse cardiovascular events compared with the general population^4^. The causes of the high prevalence of CVD in firefighter groups can be attributed to physical strain and stress, poor diet, and lifestyle factors, including long hours and shift work, lack of access to healthcare, and exposure to smoke and chemicals^3^. Firefighters are exposed to heavy metals via multiple routes^6^, which might increase the risk of CVD, including hypertension.

Heavy metals, such as lead (Pb), cadmium (Cd), and mercury (Hg), along with polycyclic aromatic hydrocarbons (PAH), are among the specific hazardous substances present in smoke and chemical emissions. Exposure to heavy metals, such as Pb, Cd, and Hg, is associated with the development of metabolic syndrome (MetS)^7–10^. MetS, a risk factor for CVD, comprises a cluster of metabolic abnormalities and is a combination of several factors, including obesity, high blood pressure, high blood sugar levels, elevated levels of triglycerides, and low levels of high-density lipoprotein (HDL) cholesterol^11–13^. Ghaedrahmat et al. reported a positive relationship between Cd and Pb levels and waist circumference (WC) in the Hoveyzeh cohort^8^. Park et al. reported that blood Pb levels were positively correlated with systolic and diastolic blood pressure, WC, fasting blood glucose, and triglyceride levels, as well as positively associated with HDL cholesterol in the Korean population^9^. The findings of the meta-analysis by Xu et al. also revealed that Cd levels were higher and Pb levels were higher in populations with MetS than those in heavy metals^10^. However, few studies have linked exposure to heavy metals and MetS in an occupational population, including firefighters. Jeon et al. demonstrated that blood Cd levels were significantly associated with a 4.3-fold increased risk of hypertension after adjusting for age, education level, work department, and work schedule^14^.

Despite their increased exposure to environmental stressors, including heavy metals, studies demonstrating the association between heavy metals and MetS in firefighters are limited. Furthermore, we lack comprehensive research on the association between heavy metals and MetS and its components in the firefighter population. Therefore, we aimed to investigate the relationship between blood Pb and Cd concentrations and MetS, including its components, such as central obesity, hypertriglyceridemia, low HDL, hypertension, and hyperglycemia, among Korean firefighters.

## Results

Of the 965 participants in this study, 190 (19.6%) had MetS. Table 1 presents the characteristics of the study population. The total population had a mean age of 42.0 ± 10.7 years. The mean age was higher in the MetS group (48.7 ± 8.5 years) than in the control group (40.4 ± 10.5 years). The geometric means (GMs) of blood Pb and Cd levels were 1.85 μg/dL (95% confidence interval [CI]: 1.80, 1.89) and 0.67 μg/dL (95% CI 0.64, 0.69), respectively, for the total population, and 2.02 μg/dL (95% CI 1.91, 2.13) and 0.82 μg/dL (95% CI 0.76, 0.87), respectively, for the MetS group. The corresponding values for the control group were 1.81 μg/dL (95% CI 1.76, 1.85) and 0.63 μg/dL (95% CI 0.61, 0.66). Approximately one-fourth of the MetS group were non-smokers, and the majority (60.5%) were ex-smokers. In the MetS group, the percentage of ex-drinkers was higher than that of current drinkers. Approximately three-fourths of the population exercised three days or less during the previous seven days, regardless of their MetS status. Approximately two-thirds of the population reported rotating duty. Fire-control workers accounted for the largest proportion (45.3%) of the study population, followed by paramedics and rescue workers (19.4%) and office administrators (17.7%).

**Table 1:** Characteristics of the study population.

Characteristics	Total (n = 965)	Metabolic syndrome (n = 190 )	Non-metabolic syndrome (n = 775)	*P*-value
Age (year)	42.03 ± 10.66	48.66 ± 8.51	40.41 ± 10.51	< 0.0001
BMI (kg/m^2^)	24.92 ± 2.62	26.83 ± 2.42	24.45 ± 2.45	< 0.0001
Triglyceride (mg/dL)	141.83 ± 88.38	225.23 ± 119.16	121.39 ± 64.30	< 0.0001
HDL cholesterol (mg/dL)	50.79 ± 11.97	41.89 ± 8.88	52.98 ± 11.62	< 0.0001
Fasting glucose (mg/dL)	90.35 ± 13.83	100.56 ± 20.92	87.85 ± 9.97	< 0.0001
SBP (mmHg)	127.54 ± 12.25	132.23 ± 13.26	126.39 ± 11.72	< 0.0001
DBP (mmHg)	80.60 ± 9.67	84.89 ± 10.15	79.55 ± 9.25	< 0.0001
Waist (cm)	86.45 ± 7.09	92.59 ± 5.85	84.95 ± 6.54	< 0.0001
Heavy metal				
Lead (µg/dL)	1.85 (1.80, 1.89)	2.02 (1.91, 2.13)	1.81 (1.76, 1.85)	0.0001
Cadmium (µg/dL)	0.67 (0.64 ,0.69)	0.82 (0.76,0.87)	0.63 (0.61, 0.66)	< 0.0001
Smoking				0.0057
None	334 (34.61)	48 (25.26)	286 (36.90)	
Ex-smoker	529 (54.82)	115 (60.53)	414 (53.42)	
Smoker	102 (10.57)	27 (14.21)	75 (9.68)	
Drinking				0.0060
None	70 (7.25)	21 (11.05)	49 (6.32)	
Ex-drinker	597 (61.87)	100 (52.63)	497 (64.13)	
Drinker	298 (30.88)	69 (36.32)	229 (29.55)	
Exercise for 7 days				0.27
None	156 (16.17)	41 (21.58)	115 (14.84)	
1 day	120 (12.44)	22 (11.58)	98 (12.65)	
2 days	195 (20.21)	38 (20.00)	157 (20.26)	
3 days	224 (23.21)	45 (23.68)	179 (23.10)	
4 days	102 (10.57)	16 (8.42)	86 (11.10)	
5 days	92 (9.53)	12 (6.32)	80 (10.32)	
6 days	20 (2.07)	3 (1.58)	17 (2.19)	
7 days	56 (5.80)	13 (6.84)	43 (5.55)	
Shift duty				< 0.0001
Yes	742 (76.89)	125 (65.79)	617 (79.61)	
No	223 (23.11)	65 (34.21)	158 (20.39)	
Main duty position				< 0.0001
Newly recruited* workers	96 (9.95)	6 (3.16)	90 (11.61)	
Fire-control workers	437 (45.28)	89 (46.84)	348 (44.90)	
Paramedics and rescue workers	187 (19.38)	24 (12.63)	163 (21.03)	
Office administrators	171 (17.72)	44 (23.16)	127 (16.39)	
Retirees	74 (7.67)	27 (14.21)	47 (6.06)	

* Those who completed National Fire Service Academy within one year or are undertaking the training.

BMI, body mass index; SBP, systolic blood pressure; DBP, diastolic blood pressure.

Data are represented as means ± standard deviations, geometric means (95% confidence interval), and numbers (%).

Blood Pb and Cd levels were positively associated with MetS in the linear regression results (Model 1) and were statistically significant. Significant associations were observed between blood Cd levels and MetS in Models 2 and 3 (Table 2). No significant associations were observed between blood Pb and Cd levels and the individual components of MetS in Models 2 and 3.Blood Cd concentrations and MetS were positively associatedin the non- and ex- smokers’ groups (OR = 1.58; 95% CI 1.03, 2.43) (Table 3). However, no such associations were observed in smokers. Additionally, positive associations were observed between blood Cd levels and MetS in the non- and ex-drinkers’ groups (OR = 1.77; 95% CI 1.06, 2.94), as well as in the individuals aged over 40 years (OR = 1.77; 95% CI 1.10, 2.86).

**Table 2:** Risk of metabolic syndrome associated with heavy metals in the study population.

	No. of cases (%)	Model 1 OR (95% CI)	Model 2 OR (95% CI)	Model 3 OR (95% CI)
Lead				
Metabolic syndrome	190 (19.65)	2.35 (1.51,3.64)	1.10 (0.65,1.87)	1.14 (0.67,1.94)
Central obesity	297 (30.77)	1.46 (1.00,2.13)	0.81 (0.46,1.42)	0.80 (0.45,1.41)
Hypertriglyceridemia	355 (36.79)	2.10 (1.45,3.03)	1.24 (0.82,1.88)	1.34 (0.88,2.03)
Low HDL	199 (20.62)	1.65 (1.07,2.53)	1.01 (0.63,1.62)	1.04 (0.65,1.67)
Hypertension	243 (25.18)	1.49 (1.00,2.22)	1.02 (0.66,1.57)	1.09 (0.71,1.69)
Hyperglycemia	135 (13.99)	2.30 (1.40,3.80)	1.04 (0.60,1.80)	0.99 (0.56,1.73)
Cadmium				
Metabolic syndrome	190 (19.65)	2.59 (1.87,3.60)	1.61 (1.06,2.43)	1.62 (1.07,2.46)
Central obesity	297 (30.77)	1.58 (1.22,2.06)	1.35 (0.88,2.07)	1.28 (0.83,1.99)
Hypertriglyceridemia	355 (36.79)	1.98 (1.53,2.57)	1.30 (0.95,1.77)	1.33 (0.97,1.82)
Low HDL	199 (20.62)	1.90 (1.39,2.58)	1.32 (0.93,1.87)	1.34 (0.94,1.92)
Hypertension	243 (25.18)	1.11 (0.85,1.45)	0.80 (0.58,1.10)	0.81 (0.59,1.12)
Hyperglycemia	135 (13.99)	2.20 (1.52,3.17)	1.23 (0.80,1.88)	1.20 (0.78,1.84)

OR, odd ratio; CI, confidence interval; HDL, high-density lipoprotein.

Model 1: metabolic syndrome/ Individual component of metabolic syndrome = heavy metal.

Model 2: metabolic syndrome/ Individual components of metabolic syndrome = heavy metal + age + BMI + smoking + drinking.

Model 3: metabolic syndrome/ Individual components of metabolic syndrome = heavy metal + age + BMI + smoking + drinking + exercise + shift duty + main duty position.

**Table 3:** Stratified analysis of metabolic syndrome associated with heavy metals in the study population.

	No. of cases (%)	Lead OR (95% CI)	Cadmium OR (95% CI)
Smoker	27 (26.47)	1.14 (0.21,6.28)	2.24 (0.50,10.08)
Non-smoker & Ex-smoker	163 (18.89)	1.15 (0.66,2.02)	1.58 (1.03,2.43)
Drinker	69 (23.15)	1.88 (0.76,4.68)	1.43 (0.66,3.10)
Non-drinker & Ex-drinker	121 (18.14)	0.89 (0.46,1.74)	1.77 (1.06,2.94)
Under 40 years old	38 (8.70)	1.25 (0.38,4.13)	2.04 (0.84,4.98)
Over 40 years old	152 (28.79)	1.31 (0.73,2.34)	1.77 (1.10,2.86)
Shift duty	125 (16.85)	0.99 (0.53,1.87)	1.66 (1.00,2.78)
Non-shift duty	65 (29.15)	1.69 (0.63,4.58)	1.51 (0.74,3.08)
Fire-control workers	89 (20.37)	0.90 (0.43,1.88)	1.25 (0.69,2.26)
Paramedics and rescue workers	24 (12.83)	1.13 (0.25,5.08)	1.08 (0.31,3.73)
Office administrators	44 (25.73)	2.40 (0.62,9.39)	3.85 (1.42,10.39)

OR, odds ratio; CI, confidence interval.

Further stratified analysis by shift and main duty position revealed a positive association between Cd exposure and shift duty; however, this difference was not statistically significant (Table 3). Nonetheless, in the office administration group, we observed a significant association between Cd exposure and MetS (OR = 3.85; 95% CI 1.42, 10.39).

## Discussion

We investigated the associations between Pb and Cd levels and MetS among Korean firefighters and observed that Cd levels are significantly associated with a 1.6-fold increased risk of MetS among the Korean firefighter population. Notably, this association remained significant among non-smokers, ex-smokers, non-drinkers, firefighters aged 40 years or older, firefighters on shift duty, and office administrators.

The observed association between blood Cd levels and MetS is consistent with the findings of previous studies. Some studies have reported a positive association between blood Cd levels and blood pressure^15,16^. Notably, Eum et al. suggested that Cd exposure may increase blood pressure and the risk of hypertension in the general adult Korean population^17^. Cd exposure can also lead to glucose disturbances and an increased risk of diabetes, as well as alter lipoproteins through the mediation of oxidative stress and inflammation^18–22^. Collectively, these findings support the association between Cd exposure, and MetS observed in our study. In contrast, we did not find an association between Pb levels and MetS. The Pb levels in our study were likely not sufficiently high to detect an association with MetS. For instance, Kristal-Boneh et al. reported a Pb concentration of 42.3 μg/dL, whereas a level of 2.02 μg/dL was observed in our study^23^. Moon et al. also reported the association between blood Pb and MetS in the Korean population with a slightly higher concentration than that observed in our study^24^.

Only office administrators exhibited a significant association between blood Cd levels and MetS. Although we assumed that fire-control workers were likely to be exposed to heavy metals. We observed no significant association between blood Cd levels and MetS in this group. We speculated that job type might minimally affect the blood level of Cd and that other exposures, such as dietary intake, may be more strongly related to the level of Cd and the risk of MetS. Notably, the blood Cd levels did not significantly differ among fire-control workers (GM, 0.64 μg/dL, 95% CI 0.60, 0.67), paramedics and rescue workers (GM, 0.68 μg/dL, 95% CI 0.63, 0.73), or office administrators (GM, 0.73 μg/dL, 95% CI 0.67, 0.79) in our firefighter population. On the other hand, the proportion of MetS in office administrators (36.5%) was higher than those in paramedics and rescue workers (12.8%) and fire-control workers (20.4%). It is possible that office administrators are more likely to have MetS due to sedentary job duties. The blood levels of heavy metals may not sufficiently reflect acute exposure to heavy metals via inhalation among fire-control workers. Future studies must be conducted to collect and track airborne heavy metal exposures in this population. Furthermore, the null association between heavy metal exposures and MetS among fire-control workers may be explained by the healthy worker effect. Myers et al. reported that fitness could be strongly associated with MetS^25^. Currently, in Korea, firefighters are required to undergo annual fitness tests, which might increase physical capacity and lead to a low prevalence of MetS^27^.

Positive associations between Cd and MetS were observed among individuals over 40 years old, which is consistent with the fact that CVD is an age-dependent disease with a higher risk in middle-aged individuals. A significant association between Cd and MetS was also observed in non-smokers and ex-smokers, as smoking is one of the exposure factor to Cd^28–30^. However, the lack of data collection on the main Cd exposure pathways, including food intake such as rice, vegetables, and potatoes^31^, may have affected the results. A significantly higher association between Cd and MetS was also observed in groups of non-drinkers and ex-drinkers. However, few studies have investigated the effects of alcohol consumption on Cd exposure. This can be explained by the fact that foods consumption may have affected the results. Therefore, future studies must include dietary information to obtain more concrete results. Canuto et al. observed an association between Cd levels and MetS in shift-duty workers, as changes in eating habits resulting from shift work may affect this association^32^. However, we lacked food intake information for the participants; therefore, dietary habits in the firefighter group must also be investigated in further research.

This study has some limitations. First, we conducted a cross-sectional study of baseline cohort data to investigate the association of blood Cd and Pb levels with MetS and its components. We cannot exclude the possibility of reverse causation. Future follow-up studies are required to estimate the risk of developing MetS associated with heavy metal exposures. Second, this study did not consider confounders such as dietary intake and exposure period of duty position. Third, heavy metals (Cd and Pb) exposure was only evaluated once using blood samples, which may have been affected by smoking^33^. Nonetheless, we conducted a stratified analysis by smoking status and observed a positive association between Cd and MetS in non-smokers and ex-smokers.

In summary, our findings reveal a significant association between blood Cd concentrations and an increased risk of MetS among firefighters, especially among non-smokers and ex-smokers, non-drinkers and ex-drinkers, firefighters aged 40 years or older, and office administrators. However, this cross-sectional study cannot exclude the possibility of reverse causation, and dietary intake data were unavailable. In addition, heavy metals exposure status was evaluated only once, which may have been affected by smoking. Further large-scale longitudinal studies are required to overcome these limitations and provide a more comprehensive understanding of the pathophysiological role of occupational exposure to Cd in firefighters with MetS.

## Methods

### Study population

The Firefighter Research on the Enhancement of Safety and Health (FRESH) Cohort Study collected data from 1,022 firefighters between 2016 and 2017. Participants were recruited from hospitals in Seoul (Severance Hospital), Wonju (Severance Christian Hospital), and Jinju (Gyeongsang National University Hospital)^34^. To ensure data quality, we excluded participants with missing data, and only male firefighters were included, as only 44 female firefighters participated. The final analysis included 965 participants. Duty positons of the study population included newly recruited workers, fire-control workers, paramedics and rescue workers, office administrators, and retirees. The Median dispatch frequencies for the past one month prior to enrollment were 15.0 times (25–75%, 5.0–40.0) for newly recruited workers, 15.0 times (25–75%, 30.0–8.0) for fire-control workers, 40.0 times (25–75%, 80.0–20.0) for paramedics and rescue workers, 0.0 times (25–75%, 1.0–0.0) for office administrators, and 0.0 times (25–75%, 6.0–0.0) for retirees.

### Definition of MetS

In accordance with the 2005 revised National Cholesterol Education Program-Adult Treatment Panel III criteria and Korean Society for the Study of Obesity criteria for WC^1,35^, we determined the components of MetS as follows: (1) central obesity (WC ≥ 90), (2) hypertriglyceridemia (≥ 150 mg/dL) or current use of anti-dyslipidemia medication, (3) low HDL cholesterol (< 40 mg/dL) or current treatment for lipid abnormality, (4) elevated fasting glucose levels (≥ 100 mg/dL) or current use of anti-diabetic medications, and (5) elevated blood pressure (average systolic blood pressure ≥ 130 mmHg and diastolic blood pressure ≥ 85 mmHg) or current use of blood pressure medication. MetS was defined as the presence of at least three of the five components mentioned above.

### Data collection

Self-reported questionnaires were administered along with physical examinations to collect information on demographics, working status, lifestyle, and physical activity, as per a standardized protocol. Blood samples were obtained after an eight-hour fast. The enzymatic method was used to analyze blood glucose level. Blood HDL-cholesterol and triglycerides levels were analyzed using the colorimetry method. Serum heavy metals (Pb and Cd) were analyzed using inductively coupled plasma-mass spectrometry. All blood analyses were conducted at a single laboratory (Seoul Clinical Laboratories Co.,Ltd., in Seoul, Korea).

Blood pressure was measured three times using a digital blood pressure monitor on the right arm, while the participants were seated after a certain rest period, and the average of the measurements was used. WC was measured halfway between the costal margin and the iliac crest. Height and weight were measured, while the participants wore lightweight clothing without shoes. Information on the physician-diagnosed or medical history of MetS components-related diseases (hypertriglyceridemia, hyperlipidemia, hyperglycemia, and hypertension) was collected using self-reported questionnaires.

The covariates included age, height, and weight for body mass index (BMI, kg/m^2^); medical history; smoking status (smoker, ex-smoker, and non-smoker); alcohol consumption (drinker, ex-drinker, and non-drinker); exercise status (frequency of moderate-level exercise for the last 7 days); shift duty (yes or no); and main duty positions (newly recruited workers, fire-control workers, paramedics and rescue workers, office administrators, and retirees). The BMI was calculated using the height and weight data. The work status, shift duty, and main duty position data were collected using self-reported questionnaires. Newly recruited workers were those who had completed the National Fire Service Academy within one year or were currently undergoing training.

### Statistical analysis

Blood Cd and Pb levels were expressed as geometric means (GM) with 95% CI and were log-transformed to analyze their distributions because they were positively skewed. Descriptive statistics were used to express means with standard deviations and frequency distributions for continuous and categorical variables, respectively. T-tests and chi-square analyses were conducted to analyze their statistical significance.

The associations between blood Cd and Pb levels and MetS in Korean male firefighters were examined using three logistic regression models. Model 1 was not adjusted for any covariates. Model 2 was adjusted for age, BMI, smoking, and alcohol drinking. Model 3 included the Model 2 variables as well as exercise, shift duty, and main duty position. The associations between blood Cd and Pb levels and each of the MetS components were also examined. Stratified analyses were conducted according to smoking status, drinking status, age, shift duty, and main duty position (fire-control workers, paramedics and rescue workers, and office administrators) to determine the characteristics of the personal habits and job positions that might be related to the association between MetS and heavy metals The analyses were performed using SAS version 9.4 (SAS Institute, Cary, NC, USA), and statistical significance was set at P < 0.05.

### Ethical approval and consent of participate

All data were collected after approval from the Institutional Review Board of the Yonsei University College of Medicine (4-2016-0187) and informed consent was obtained from all participants. The Study was conducted in accordance with the Declaration of Helsinki.

## Data Availability

The datasets were generated during the FRESH cohort study and are not publicly available due to personal information of the research participants but are available from the corresponding author upon reasonable request.
